# Phylogenetic and genetic characterization of *Treponema pallidum* strains from syphilis patients in Japan by whole-genome sequence analysis from global perspectives

**DOI:** 10.1038/s41598-021-82337-7

**Published:** 2021-02-04

**Authors:** Shingo Nishiki, Kenichi Lee, Mizue Kanai, Shu-ichi Nakayama, Makoto Ohnishi

**Affiliations:** 1 Department of Bacteriology I, National Institute of Infectious Diseases, 1-23-1 Toyama, Shinjuku-ku, Tokyo, 162-8640 Japan; 2 Division of Environmental and Preventive Medicine, Department of Social Medicine, Graduate School of Medicine, Tottori University, 86 Nishi-machi, Yonago, Tottori 683-8503 Japan; 3 Osaka City Public Health Office, 1-2-7-1,000 Asahi-cho, Abeno-ku, Osaka, Osaka 545-0051 Japan

**Keywords:** Genome assembly algorithms, Computational biology and bioinformatics, Evolution, Molecular evolution, Microbiology, Bacteria, Bacterial genomics

## Abstract

Japan has had a substantial increase in syphilis cases since 2013. However, research on the genomic features of the *Treponema pallidum* subspecies *pallidum* (TPA) strains from these cases has been limited. Here, we elucidated the genetic variations and relationships between TPA strains in Japan (detected between 2014 and 2018) and other countries by whole-genome sequencing and phylogenetic analyses, including syphilis epidemiological surveillance data and information on patient sexual orientation. Seventeen of the 20 strains in Japan were SS14- and the remaining 3 were Nichols-lineage. Sixteen of the 17 SS14-lineage strains were classified into previously reported Sub-lineage 1B. Sub-lineage 1B strains in Japan have formed distinct sub-clusters of strains from heterosexuals and strains from men who have sex with men. These strains were closely related to reported TPA strains in China, forming an East-Asian cluster. However, those strains in these countries evolved independently after diverging from their most recent common ancestor and expanded their genetic diversity during the time of syphilis outbreak in each country. The genetic difference between the TPA strains in these countries was characterized by single-nucleotide-polymorphism analyses of their penicillin binding protein genes. Taken together, our results elucidated the detailed phylogenetic features and transmission networks of syphilis.

## Introduction

*Treponema pallidum* subspecies *pallidum* (TPA) is the etiologic agent of syphilis. An estimated 6 million new cases are diagnosed globally every year, with the majority in low- and middle-income countries^1^. However, a resurgence of syphilis has been reported in developed countries worldwide beginning in the twenty-first century^2^, including a substantial increase in syphilis cases in Japan since 2013^3^.

To date, most molecular studies of TPA have been carried out by PCR amplification of several TPA genetic loci^4,5^. These have identified some epidemiological features related to the subtype and to the prevalence of macrolide resistance in strains in different countries^6^. Although whole-genome sequence (WGS) analyses have provided phylogenetic information on TPA strains in several countries^7–14^, those data have not necessarily been accumulated enough for comprehension of the overall aspects of the TPA circulations in the world.

The annual number of syphilis cases in Japan exceeded 7000 in 2018, which was the highest annual number of syphilis cases in 50 years^15^. However, research on the genomic features of the TPA strains from these cases has not been reported. Therefore, we conducted molecular typing and WGS analyses of TPA strains detected between 2014 and 2018 in Tokyo and Osaka prefectures which accounted for about 40% of all the cases in Japan. Then, we reconstructed the phylogenetic relationships between these TPA genomes and the publicly available genomes in other countries. Moreover, the genetic variations and global relationships of TPA strains, and the transmission networks of syphilis have been discussed using syphilis epidemiological surveillance data and patient gender and sexual orientation data.

## Results

### Molecular typing

For this study, 139 *Treponema pallidum* genomes that satisfied the criteria described in “Methods” were used for phylogenetic analysis: 20 genomes of TPA strains detected in Japan and, in GenBank, 117 genomes of TPA strains and 2 genomes of *Treponema pallidum* subspecies *pertenue* (TPE) strains. The patient and clinical information, and molecular typing results of the 20 TPA strains detected in Japan are shown in Table 1. These 20 TPA strains were from 9 heterosexual males, 6 men who have sex with men (MSM), 4 heterosexual females, and 1 case with missing data on gender and sexual orientation. These 20 were selected as follows. We identified 49 TPA strains among the samples collected from patients in Japan with suspected syphilis between 2014 and 2018, that were *Treponema pallidum* DNA-positive, and had been fully typed by the Enhanced Centers for Disease Control and Prevention (ECDCT) typing^4^. These 49 strains were analyzed by WGS analysis, and 20 strains satisfied the criteria of at least 90% coverage of the Nichols genome and a minimum of 10 reads in the WGS analysis (see “Methods” for details). 117 previously published treponemal genomes met the same criteria and were included in our study. According to the ECDCT system, all the strains from heterosexual male and heterosexual female cases were the 14d/f subtype, and the strains from MSM cases included the predominant 14d/f subtype and several other subtypes (Table 1).

**Table 1:** Clinical information and molecular typing results of 20 Japanese *Treponema pallidum* subspecies *pallidum* strains.

Sample ID	Sample type	Year detected	Detection location	Patient gender	Patient sexual orientation	Genome coverage (%)	Lineage	Sub-lineage	*arp* type	*tpr* type	*tp0548* type	Macrolide sensitivity
14B001UK	Swab	2014	Tokyo	NA	NA	93.7	Nichols	–	14	d	c	S
15A011MM	Swab	2015	Tokyo	Male	MSM	98.4	SS14	1B	14	e	f	S
15A019HM	Swab	2015	Tokyo	Male	HM	98.4	SS14	8	14	d	f	R
16A013MM	Swab	2016	Tokyo	Male	MSM	91.1	SS14	1B	14	d	f	R
17A003MM	Swab	2017	Tokyo	Male	MSM	98.3	Nichols	–	14	b	c	S
17A014MM	Swab	2017	Tokyo	Male	MSM	97.3	SS14	1B	14	d	f	S
17A017MM	Swab	2017	Tokyo	Male	MSM	98.3	SS14	1B	14	d	f	R
17A021MM	Swab	2017	Tokyo	Male	MSM	98.3	Nichols	–	12	a	c	R
17C005HM	Swab	2017	Tokyo	Male	HM	90.8	SS14	1B	14	d	f	R
17C009HM	Swab	2017	Tokyo	Male	HM	96.0	SS14	1B	14	d	f	R
17D002HM	Swab	2017	Osaka	Male	HM	98.3	SS14	1B	14	d	f	R
17D006HM	Swab	2017	Osaka	Male	HM	92.7	SS14	1B	14	d	f	R
17D009HM	Swab	2017	Osaka	Male	HM	96.0	SS14	1B	14	d	f	R
17E002HF	Swab	2017	Tokyo	Female	HF	95.2	SS14	1B	14	d	f	R
18C004HM	Swab	2018	Tokyo	Male	HM	94.5	SS14	1B	14	d	f	R
18D001HM	Swab	2018	Osaka	Male	HM	95.2	SS14	1B	14	d	f	R
18D002HM	Swab	2018	Osaka	Male	HM	96.6	SS14	1B	14	d	f	R
18E001HF	Swab	2018	Tokyo	Female	HF	97.8	SS14	1B	14	d	f	R
18E002HF	Swab	2018	Tokyo	Female	HF	98.3	SS14	1B	14	d	f	R
18E016HF	Swab	2018	Tokyo	Female	HF	98.2	SS14	1B	14	d	f	R

NA, data not available; MSM, men who have sex with men; HF, heterosexual female; HM, heterosexual male; S, sensitive; R, resistant.

### Phylogenetic and temporal analyses

**Figure 1:**
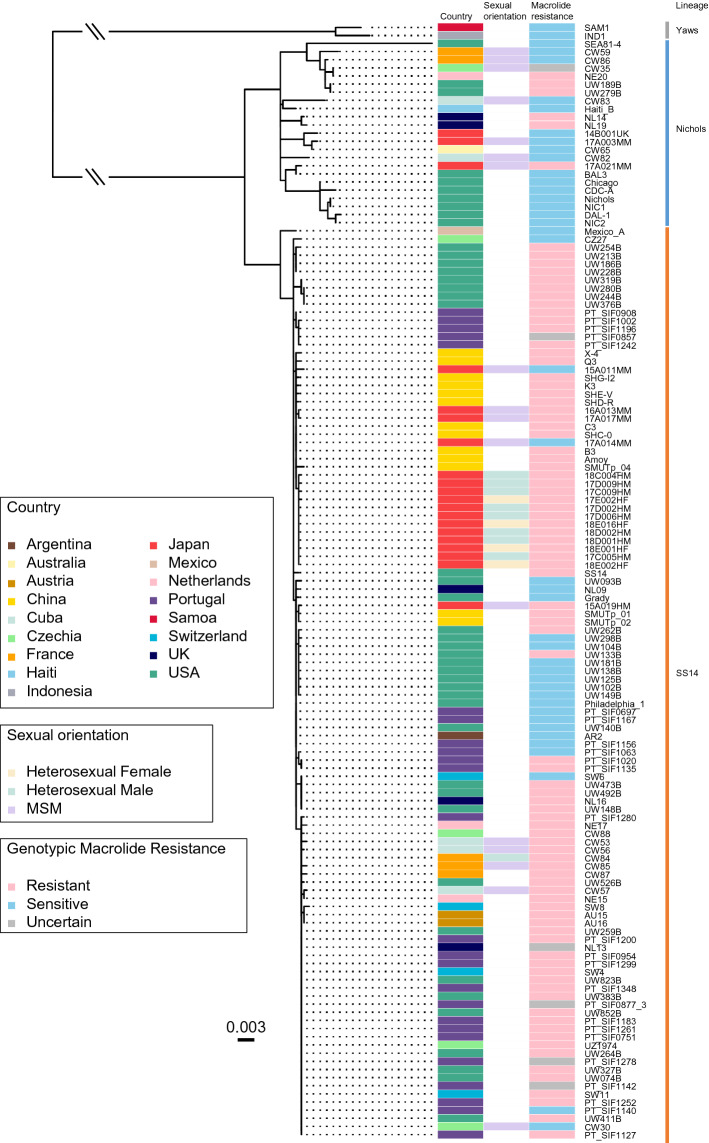
Maximum likelihood phylogenetic analysis of 135 *Treponema pallidum* subspecies *pallidum* and 2 *Treponema pallidum* subspecies *pertenue* genomes. The boxes in the columns show (from the left) for each strain: its lineage, its country of origin, sexual orientation of the patient, and its macrolide resistance genetic profile. For macrolide resistance, if either the A2058G or A2059G mutation in the 23S rRNA genes was present, the strain was classified as resistant. If mixed alleles were detected in these sites, the strain was classified as ‘‘uncertain’’. The scale represents the number of substitutions per site.

**Figure 2:**
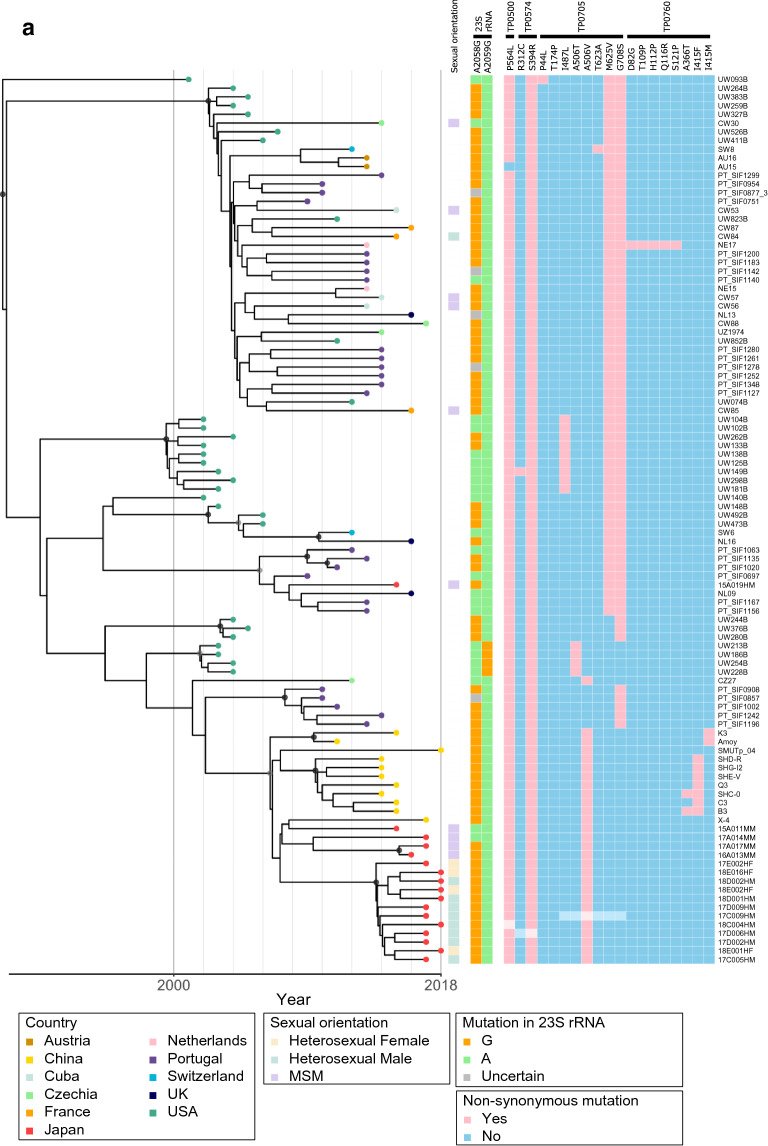

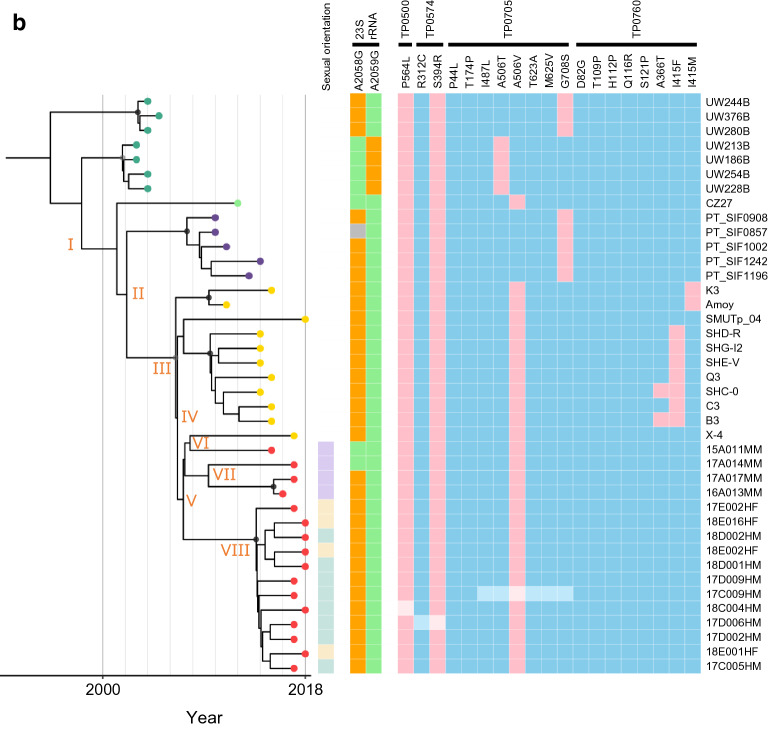
Bayesian maximum credibility phylogenetic analysis of 102 clinical *Treponema pallidum* subspecies *pallidum* genomes. (**a**) Time scaled phylogenetic tree of the TPA genomes in this study. (**b**) Expanded view of East Asian cluster-related node. The colored nodes represent the country of origin of the TPA genomes. The branch point nodes are shaded to indicate the posterior support (black ≥ 96%, dark grey ≥ 91%). The boxes on the right indicate the sexual orientation of the patient, the resistant mutations in the 23S rRNA genes, and the non-synonymous mutations in the penicillin-binding protein genes as shown in the legends. For the 23S rRNA genes, orange and green colors indicate the resistant-type and wild-type sites, respectively. For the penicillin-binding protein genes, pink and blue colors indicate the mutant and wild-type sites, respectively. The boxes with light-colored squares are single nucleotide polymorphism (SNP) sites that were not detected in the draft genome, and were determined by PCR-based Sanger sequencing with the primers shown in Table S2. The divergence estimates (95% HPD) of the time of each node are shown as follows: I, 1998 (1995–2002); II, 2002 (2000–2007); III, 2006 (2003–2009); IV, 2006 (2004–2010); V, 2007 (2006–2012); VI, 2008 (2006–2012); VII, 2009 (2008–2015); VIII, 2013 (2010–2016).

A maximum likelihood phylogenetic tree was reconstructed for the 135 TPA and 2 TPE genomes (Fig. 1): these strains were from 17 countries. Of the TPA genomes, 112 (including 83 macrolide-resistant genomes) were classified as SS14-lineage and 23 (including 6 macrolide-resistant genomes) as Nichols-lineage. For the strains in Japan, 17 strains (including 15 macrolide-resistant genomes) were SS14-lineage and the other 3 strains (including 1 macrolide-resistant genome) were Nichols-lineage. Subsequently, temporal analyses were performed with clinical and low-rabbit passaged strains (Table S1). Root-to-tip regression analysis of the116 genomes did not indicate a clock-like signal (slope rate = − 0.0001, R^2^ = 0.0017). Meanwhile, clock-like signals were indicated when the root-to-tip analysis was performed separately in SS14 and Nichols. To further confirm the temporal signal in our tree, we performed date-randomization test in each lineage. The test indicated that the temporal signal observed in SS14 lineage was not obtained by chance (Fig. S1). Therefore, we performed a temporal analysis using BEAST2 program with 102 genomes of SS14 lineage (Fig. 2a).

According to previous studies^10,11^, in addition to classification in the Nichols and SS14 lineages, as the SS14-lineage can be partitioned into two lineages (SS14Ω-A and SS14Ω-B) and these two lineages can be further partitioned into Sub-lineages 2–8 (SS14Ω-A) and Sub-lineages 1A/1B (SS14Ω-B), respectively, our results were in agreement with these classifications (Fig. 2a). The most recent common ancestor (MRCA) of the SS14-lineage was estimated to be extant between 1960 and 1980 (Fig. 2a). All of the SS14-lineage strains in Japan, except one, formed an East-Asian cluster (EAC), previously named Sub-lineage 1B^11^, consisting of 16 TPA strains detected in Japan and 11 TPA strains detected in China (Fig. 2a,b). The other SS14-lineage strain detected in Japan (strain 15A019HM, Fig. 2a) was in a sub-lineage that included TPA strains in European countries, although coming from a patient lacking information on nationality and a travel history.

### Macrolide resistance and SNP analyses

With regard to the macrolide resistance mutations in Sub-lineage 1 genomes, unlike Sub-lineage 1A^11^ that contained four strains (UW186B, UW213B, UW228B, and UW254B) detected in the U.S, all of the macrolide-resistant strains in Sub-lineage 1B, the EAC, carried a single A2058G mutation in their 23S rRNA genes (Fig. 2b). In addition, 2 macrolide-sensitive strains were detected from MSM cases in Japan (derived from node V in Fig. 2b).

Various amino acid changes in the penicillin binding protein (PBP) in the *Treponema* genome have been reported^9,11,12^. Especially, Sun et al. discussed on the probable relationships between these amino acid changes among the strains in China and drug pressure of penicillin^9^. Although there has been no evidence for the hypothesis that those amino acid changes have some effect on penicillin resistance, they might be useful as phylogenetic markers. This prompted us to investigate those changes among the strains in Japan. Therefore, based on previous studies^9,11^, we selected four PBP genes (*pbp1* (TPANIC_0500), *pbp2* (TPANIC_0760), *mrcA* (TPANIC_0705)*,* and *Tp47* (TPANIC_0574)) for this study. The *mrcA* A506V mutation was a unique feature in the genomes of EAC strains and was in all the genomes of TPA strains detected in Japan and China. However, the *mrcA* A506V mutation was not found in the genomes of other sub-lineage genomes, except strain CZ27 (Fig. 2a,b). The variants associated with *pbp2* (TPANIC_0760) were only found in the genomes of TPA strains in China (Fig. 2a,b), and most (9 of 11) of these strains harbored *pbp2* (TPANIC_0760) I415F or I415M mutation. Among the EAC strains detected in Japan, no genetic difference in the four genes (i.e., *pbp1*, *pbp2*, *mrcA* and *Tp47*) was observed. In addition, among the eight Nichols-lineage strains, only one strain detected in Japan (17A021MM) carried the *pbp1* P564L mutation (Fig. 2a). This was the first example of a Nichols-lineage strain that carried this mutation.

### The genetic diversifications of TPA strains and calculations of MRCA in China and Japan

The EAC MRCA was estimated to have been extant in 2006 (95% Highest Posterior Density (HPD): 2003–2009, node III in Fig. 2b), with the TPA strains in China and Japan subsequently forming separate clusters (Fig. 2b). The MRCA of the clusters of TPA strains in China and Japan may have emerged in about 2006 (95% HPD: 2003–2009, node III in Fig. 2b) and 2007 (95% HPD: 2006–2012, node V in Fig. 2b, respectively. The 11 TPA strains in China (from 7 men, 2 women, and 2 cases with missing data)^8,9,14^ appeared to have increased their genetic diversity after the late 2000s. All but one of the TPA strains detected in Japan (15A011MM in Fig. 2a,b) formed distinct sub-clusters between strains from MSM cases and strains from heterosexual cases. Noticeably, the strain 15A011MM is very closely related to the strain in China, X-4, which was apparently separated from other strains in China (Fig. 2b).

The MRCA of the 3 TPA strains from MSM cases was likely to be extant in 2009 (2008–2015, node VII in Fig. 2b), and that of the 12 TPA strains from heterosexual cases was likely to be extant in 2013 (2010–2016, node VIII in Fig. 2b). The genomes of the TPA strains in Japan expanded their genetic diversity mainly after the mid-2010s (Fig. 2b).

**Figure 3:**
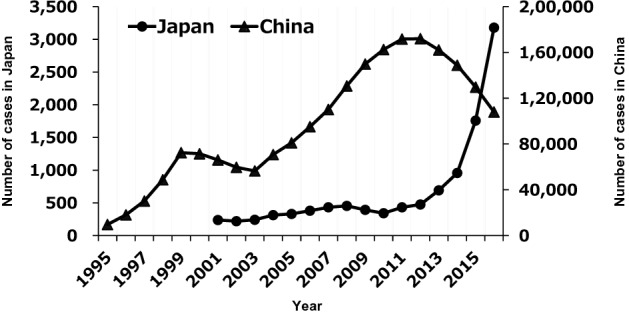
Number of primary and secondary syphilis cases reported in Japan and China, 1995–2016. The number of primary and secondary syphilis cases reported in Japan (plotted with circles, left scale) and in China (plotted with triangles, right scale). The original data were derived from The Japan National Tourism Organization (https://www.jnto.go.jp/jpn/statistics/visitor_trends/index.html).

Figure 3 shows the epidemiological trends of the primary and secondary syphilis cases reported in Japan and China between 1995 and 2016. Considering the epidemiological data and the results of the Bayesian temporal analysis for the EAC genomes, the genomes of the TPA strains in China appeared to have expanded their genetic diversity after about 2005 (Fig. 2b), which corresponded to the time of the second wave of syphilis cases in China (Fig. 3). The increase in the number of syphilis cases in Japan was after 2013 (Fig. 3), which corresponded to the time of increasing genetic diversity in TPA strains in Japan (Fig. 2b).

## Discussion

In this report, we have described the genomic and phylogenetic features of TPA strains detected in Japan compared to TPA strains detected in other countries, in particular in China. A significant feature of this study was that our analysis included information on the gender and sexual orientation of the syphilis patients from whom TPA strains had been detected.

The maximum likelihood phylogenetic analysis and the Bayesian temporal analysis of the genomes of global TPA strains in this study found similar results to previous reports in terms of lineages and sub-lineages. In those reports, most of the SS14-lineage strains in American and European countries were classified in lineages SS14Ω-A^10,11^.

The majority of TPA strains analyzed in this study in Japan (16 of 20) were classified in the SS14-lineage and formed an EAC, designated Sub-lineage 1B (in lineage SS14Ω-B) in a previous study^11^, that included strains in China. In addition to these strains, there were 3 Nichols-lineage strains and a strain belonging to another SS14 sub-lineage, previously designated as Sub-lineage 8, which contained strains in the U.S. and European countries^11^. WGS indicated an ongoing concurrent circulation of Nichols- and SS14-lineage strains in Japan, as has also been observed in several American and European countries^10,11^. Recently, four TPE strains from Japan were reported between 2014 and 2018^16^. However, the definitions of subspecies of those strains were based on the sequencing of *tp0548* and *tp0856* genes, but were not based on WGS analyses^16^. Although we could not detect any TPE strain among the strains that passed our criteria described above and in “Methods”, we have to keep monitoring the emergence and spread of TPE strains in Japan.

The EAC was composed of SS14-lineage TPA strains in China and Japan, with most TPA strains in Japan (16/17) being subtype 14d/f, which is the dominant subtype among both heterosexual and MSM cases in Japan^17^. We could not evaluate the difference between subtype 14d/f strains from MSM and heterosexual cases in our previous molecular typing studies^17,18^. The WGS study in this report elucidated the separate phylogeny of the strains from MSM and heterosexuals belonging to this same subtype (Table 1, and Fig. 2b).

These results underlined the importance of having information on gender and sexual orientation in analyzing WGS studies of TPA to comprehend the detailed aspect of the circulations of strains in the respective communities of MSM and heterosexuals.

From the geographical point of view, the experimental results for strains from heterosexual cases collected in Tokyo and Osaka prefectures were mixed, showing that genetically similar strains were circulating among heterosexuals in these two prefectures that have the largest populations in Japan.

Based on the phylogenetic analyses with the strains collected since 2011 to 2018 (Table S1, strains in China and Japan), the MRCA of the EAC strains and of strains in China appeared to emerge in 2006 (node III in Fig. 2b), followed by the MRCA of strains in Japan in 2007 (node V in Fig. 2b). Therefore, the EAC has separated into Chinese and Japanese clusters since the mid 2000s. The estimated time of emergence of the MRCA of the Chinese cluster, determined in this study, was similar to that in a recent report^11^. The genomes of TPA strains in China and Japan then expanded their genetic diversity after the late 2000s and mid-2010s, respectively, which approximately corresponded to the time each country had an increasing number of primary and secondary syphilis cases (Fig. 3). This correspondence may be consistent with the fact that TPA is an obligate human pathogen and its accumulation of SNP sites increases with time. However, for the genomes of TPA strains in China, there was a discrepancy in that there was not an increase in their genetic diversity from the mid-1990s to the early 2000s (Fig. 2b), although the onset of the increase in the number of cases was in the late 1990s (Fig. 3). This fact may be attributed to detection bias owing to a limited number of samples included in this study. The simplest explanation is that genomes of TPA in China during the early stage of the syphilis outbreak had not been collected or analyzed systematically enough.

Since contagious pathogens can cross borders, the EAC may be an example of cross-border propagation of TPA strains between Japan and China. Although the cause of the current large syphilis outbreak in Japan may be uncertain, an increase in the number of travelers from China to Japan has been noted. The Japan National Tourism Organization (https://www.jnto.go.jp/jpn/statistics/visitor_trends/index.html) has reported that the number of Chinese travelers to Japan has increased rapidly since 2014, which corresponded with the onset of the recent syphilis outbreak (Fig. 3) mainly among heterosexuals in Japan. However, our results indicated that the MRCA of the TPA strains in the sub-cluster formed by the strains from heterosexuals in Japan was likely to be extant in 2013 (node VIII in Fig. 2b). In addition, based on their phylogeny, the MRCA of TPA strain 15A011MM and the strains from heterosexuals in Japan may have been extant in 2007 (node V in Fig. 2b). Therefore, we consider the hypothesis of a connection between Chinese tourists and the syphilis outbreak in Japan controversial, although our interpretation is based on a limited number of samples and on values with a wide (6–7 year) 95% HPD (Fig. 2b).

The results of this study indicated several features about the genetic variants of some TPA genes. First, macrolide resistance mutations in TPA might be reversible, although there have been no data indicating reversion to the wild-type allele. As all the strains in Japan in Fig. 2b were phylogenetic branches from node V, it seems more likely that macrolide resistance was extant at the time node V formed, rather than that most of the strains in Fig. 2b independently mutated to macrolide resistance during the relatively short time after node V formed. Macrolide resistance mutations have been noted previously to have strong stability^11,19^, but existence of 2 macrolide-sensitive TPA strains in Japan (Fig. 2b) implied that there might have been a reverse mutation because of a fitness cost associated with carrying those mutations. However, a fitness cost- hypothesis contradicts the fact that most of the strains in Japan depicted in Fig. 2b still keep the resistance mutations. So, we could not exclude the possibility that the apparent ‘reverse mutations’ occurred independent of the putative fitness cost during the diversifications as other general SNPs did and have been kept under the absence of the drug. This scenario might meet the fact that azithromycin is no longer recommended for treatment to syphilis in most of the countries in the world.

Second, all the macrolide-resistant strains in Japan in this study carried the A2058G mutation in the 23S rRNA gene, but not the A2059G mutation. This was in agreement with the results of our previous study of over 100 strains in Japan^17,18^.

Third, in this study, the *mrcA* A506V mutation, which was considered to be unique to strains in China^9,11^, was also observed in EAC strains in Japan. However, some mutations in the *pbp2* gene (TPANIC_0760) were not identified in any of the strains in Japan, while most of the strains (9 of 11) in China harbored at least 1 mutation in this gene. Of the 3 SNPs (i.e., A366T, I415F, and I415M) in the TPANIC_0760 gene, only I415F has been suggested to have a deleterious effect on the protein’s structural flexibility or its binding constant for substrate stability^9^. However, the effect of these SNPs on the possible generation of the penicillin-resistance is not known at present, because there has been no documentation of penicillin-resistant TPA strain so far, although penicillin has been used extensively to treat syphilis for more than 70 years.

Apart from that, in this study, the separation between the clusters of TPA strains in China and in Japan shown by the phylogenetic analyses was found, for most of the strains, in the SNP analyses of the TPANIC_0760 gene, although there were 2 exceptional strains in China that had no mutation in this gene (SMUTp_04 and X-4, Fig. 2b). In this context, it is noticeable that the strain X-4 was one of those 2 exceptional strains in China, and was phylogenetically separated from other strains in China (Fig. 2b). This strain was, rather, very closely related to a Japanese strain 15A011MM forming a small sub-cluster which was branch from node VI (Fig. 2b). These lines of information implied that this small sub-cluster might reflect the limited case(s) of direct international spread of TPA between China and Japan by the ‘Japanese type’ strain(s) (with TPANIC_0705 A506V mutation and without any mutation in TPANIC_0760 gene) in the recent year.

Finally, this study confirmed a Nichols-lineage strain carrying the *pbp1* P564L mutation. This SNP has been commonly observed in, and limited to, the genomes of SS-14 strains^9,11^. The study reported here strongly suggested that TPA strains in any lineage could carry mutations in *pbp1.*

In conclusion, most of the TPA strains in Japan in this study had a close relationship to TPA strains in China, forming the EAC. The MRCA of the EAC is likely to have become extant about 2006. The TPA strains in China and Japan subsequently formed a separate cluster in each country in about 2007. The genomes of the TPA strains in each country then expanded their phylogenetic diversity during the time that country had an increasing number of syphilis cases. In addition, phylogenetic analysis showed that TPA strains from MSM cases in Japan clustered separately from strains from heterosexual cases. These findings, within the context of the recent global resurgence of syphilis, provide a better understanding of the phylogenetic features and transmission networks of syphilis, both domestic and global.

## Methods

### Clinical samples

Samples were collected from patients with suspected syphilis between 2014 and 2018 in four clinics and one hospital in Tokyo and Osaka prefectures, along with information on each patient’s gender and self-reported sexual orientation. These information were collected voluntarily. Other information of the patients was not systematically collected. It was suggested that most of the patients were in the primary or secondary stage, although not necessarily specified. This study was approved by the institutional review board of the National Institute of Infectious Diseases (approval numbers 508 and 705), and all the experiments were performed in accordance with the directions from the review board above. The informed consent was obtained from all participants.

### Detection, DNA extraction, and molecular typing of treponemal DNA

Tris–EDTA (TE) buffer-suspensions (swab samples from genital, anal, and oral lesions) and DNA extracts (the other samples) prepared by a DNeasy Blood & Tissue Kit (Qiagen, Inc., Valencia, CA) were used as the PCR templates. The TPA *polA* and *tp47* genes-targeted PCR was performed as described previously^17,18^. When at least one amplicon of the two genes was generated, a sample was considered to contain TPA-DNA. Molecular typing according to the ECDCT system^4^ and determining the presence of the A2058G and/or A2059G mutations in one or both of the TPA 23S rRNA genes were performed by short-read mapping as described below. For the Japanese strains which showed less than genome × 50 coverage in 23S rRNA genes (Table S1), Sanger sequencing was performed and no discrepancies between the two methods were confirmed.

### Whole-genome sequence and phylogenetic analyses

For WGS analysis, we selected 49 TPA strains with complete typing results in the ECDCT system^4^, which had been previously detected in Japan. Extracted DNA of the clinical samples of these strains was amplified evenly using the Illustra GenomiPhi V2 DNA Amplification Kit (GE Healthcare UK Ltd., Buckinghamshire, UK) by the functions of random hexamer nucleotides and the bacteriophage phi-29 DNA polymerase. The amplified DNA was served for fragmentation and simultaneous transfer of the adaptor tags by transposase, followed by selective concentration of TPA genomic DNA using a SureSelect QXT Target Enrichment Kit and Custom Capture Library RNA (Agilent Technologies, Ceder Creek, TX, USA) for the TPA strain Nichols genome sequence by the hybridization to Library RNA in the tube. These procedures were all performed following the manufacturer’s directions. The pooled libraries were analyzed by multiplex paired-end sequencing (300-mer × 2) using the MiSeq System (Illumina, San Diego, CA, USA). The short-reads were mapped to the complete genome of TPA strain Nichols (NCBI accession no. NC_021490.2). The genomes of 20 of the 49 TPA strains met our criteria of at least 90% coverage of the Nichols genome and a minimum of 10 reads. In addition, published treponemal genomes that met our criteria (Table S1) were included in our analyses. These short-reads were mapped to the Nichols genome using bwasw function of bwa v.0.7.17^20^ and SNPs were identified using samtools v.1.9^21^ and VarScan v.2.4.3^22^ as previously described^23^. The default parameters were used for SNP identification except that the minimum coverage was set to ten. Repeat and recombinogenic regions were detected by MUMmer v.3.2259^24^ and gubbins^25^, respectively, and were removed from further analyses. Phylogenetic relationships were determined by reconstructing a phylogenetic tree by the maximum likelihood method using IQ-TREE with 1000 ultrafast bootstrap replicates^26^.

For temporal analyses, the Bayesian Evolutionary Analysis Sampling Tree (BEAST) v. 2.6.1 program^27^ was used. Whole genome sequences of all the TPA strains detected in Japan in this study and TPA genomes in the database with no or limited passages (Table S1) were used for this analysis as previously described^11^. Briefly, a recombination-masked SNP alignment was analyzed using a GTR4 substitution model and a strict clock model with a burnin of 10 million cycles followed by 100 million MCMC cycles. The starting molecular clock rate was set to 3.6 × 10^–4^ as described previously^11^. Maximum likelihood and BEAST trees were plotted using a ggtree and a ggplot package of R v. 3.6.2^28^. To confirm the temporal signal in our tree, we used TempEst v.1.5.3 for root-to-tip analysis and the TIPDATINGBEAST package^29^ in R for date-randomization test as previously described^11^. Twenty new datasets with randomly assigned dates were analyzed by BEAST and the results showed no evidence of temporal signal in these replicates, suggesting that the signal in our tree was not found by chance.

Macrolide resistance SNPs were determined by mapping the short reads to the Nichols genome. For identification of non-synonymous variants of penicillin binding protein (PBP) genes *pbp1* (TPANIC_0500), *pbp2* (TPANIC_0760), *mrcA* (TPANIC_0705) and *Tp47* (TPANIC_0574), the short-reads were assembled using the SPAdes v. 3.13.1^30^. The PBP gene sequences were extracted from the draft genomes. In the cases of those variants could not be determined from draft genomes, the variants were determined by PCR-based Sanger sequencing of the initial DNA samples with the primers shown in Table S2.

### Epidemiological data on syphilis cases in Japan and China

Information on syphilis cases in Japan between 2001 and 2016 was obtained from public annual reports provided by the National Institute of Infectious Diseases website^31^. Information on syphilis cases in China between 1995 and 2016 was obtained from a published article^32^. Using these data, we determined the number of primary and secondary syphilis cases, which generally indicate more recent infections and can be a more timely indication of the ongoing spread of syphilis at that time than latent syphilis or asymptomatic cases.

## Supplementary Information

Supplementary Figure S1.

Supplementary Table S1.

Supplementary Table S2.

## Data Availability

*Accession numbers* Short read data have been deposited in GenBank under accession number DRA009733.

## References

[1] Newman L (2015). Global estimates of the prevalence and incidence of four curable sexually transmitted infections in 2012 based on systematic review and global reporting. PLoS ONE.

[2] Fenton KA (2008). Infectious syphilis in high-income settings in the 21st century. Lancet Infect. Dis..

[3] Takahashi T (2018). Rapid increase in reports of syphilis associated with men who have sex with women and women who have sex with men, Japan, 2012 to 2016. Sex. Transm. Dis..

[4] Marra C (2010). Enhanced molecular typing of *Treponema pallidum*: Geographical distribution of strain types and association with neurosyphilis. J. Infect. Dis..

[5] Grillová L (2018). Molecular characterization of *Treponema pallidum* subsp. pallidum in Switzerland and France with a new multilocus sequence typing scheme. PLoS ONE.

[6] Tipple C, Taylor GP (2015). Syphilis testing, typing, and treatment follow-up: A new era for an old disease. Curr. Opin. Infect. Dis..

[7] Pětrošová H (2013). Resequencing of *Treponema pallidum* ssp. pallidum strains Nichols and SS14: Correction of sequencing errors resulted in increased separation of syphilis treponeme subclusters. PLoS ONE.

[8] Tong ML (2017). Whole genome sequence of the *Treponema pallidum* subsp. pallidum strain Amoy: An Asian isolate highly similar to SS14. PLoS ONE.

[9] Sun J (2016). Tracing the origin of *Treponema pallidum* in China using next-generation sequencing. Oncotarget.

[10] Arora N (2016). Origin of modern syphilis and emergence of a pandemic *Treponema pallidum* cluster. Nat. Microbiol..

[11] Beale MA (2019). Genomic epidemiology of syphilis reveals independent emergence of macrolide resistance across multiple circulating lineages. Nat. Commun..

[12] Pinto M (2016). Genome-scale analysis of the non-cultivable *Treponema pallidum* reveals extensive within-patient genetic variation. Nat. Microbiol..

[13] Grillova L (2019). Directly sequenced genomes of contemporary strains of syphilis reveal recombination-driven diversity in genes encoding predicted surface-exposed antigens. Front. Microbiol..

[14] Chen W (2020). Analysis of *Treponema pallidum* strains from China using improved methods for whole-genome sequencing from primary syphilis chancres. J. Infect. Dis..

[15] Fujikura H (2019). Syphilis notification rate in Japan, 2004–2018. Jpn. J. Sex. Transm. Infect..

[16] Kawahata T (2019). Bejel, a nonveneral Treponematosis, among men who have sex with men, Japan. Emerg. Infect. Dis..

[17] Kanai M (2019). Molecular typing and macrolide resistance analyses of *Treponema pallidum* in heterosexuals and men who have sex with men in Japan, 2017. J. Clin. Microbiol..

[18] Nishiki S (2020). Epidemiology, molecular strain types, and macrolide resistance of *Treponema pallidum* in Japan, 2017–2018. J. Infect. Chemother..

[19] Stamm LV (2015). Syphilis: Antibiotic treatment and resistance. Epidemiol. Infect..

[20] Li H, Durbin R (2010). Fast and accurate long-read alignment with Burrows–Wheeler transform. Bioinformatics.

[21] Li H (2011). A statistical framework for SNP calling, mutation discovery, association mapping and population genetical parameter estimation from sequencing data. Bioinformatics.

[22] Koboldt DC (2012). VarScan 2: Somatic mutation and copy number alteration discovery in cancer by exome sequencing. Genome Res..

[23] Lee K, Izumiya H, Iyoda S, Ohnishi M (2019). Effective surveillance using multilocus variable-number tandem-repeat analysis and whole-genome sequencing for enterohemorrhagic *Escherichia coli* O157. Appl. Environ. Microbiol..

[24] Kurtz S (2004). Versatile and open software for comparing large genomes. Genome Biol..

[25] Croucher NJ (2015). Rapid phylogenetic analysis of large samples of recombinant bacterial whole-genome sequences using Gubbins. Nucleic Acids Res..

[26] Nguyen L-T, Schmidt HA, von Haeseler A, Minh BQ (2015). IQ-TREE: A fast and effective stochastic algorithm for estimating maximum-likelihood phylogenies. Mol. Biol. Evol..

[27] Bouckaert R (2019). BEAST 2.5: An advanced software platform for Bayesian evolutionary analysis. PLoS Comput. Biol..

[28] R Core Team. 2019. R: a language and environment for statistical computing (R Foundation for Statistical Computing, Vienna) http://www.R-project.org/, Accessed 14 Feb 2020.

[29] Rieux A, Khatchikian CE (2017). tipdatingbeast: An r package to assist the implementation of phylogenetic tip-dating tests using beast. Mol. Ecol. Resour..

[30] Bankevich A (2012). SPAdes: A new genome assembly algorithm and its applications to single-cell sequencing. J. Comput. Biol..

[31] Infectious Disease Surveillance Center, National Institute of Infectious Diseases and Tuberculosis and Infectious Diseases Control Division, Ministry of Health, Labour and Welfare. National Epidemiological Surveillance of Infectious Diseases (NESID) Program annual reports, 2001–2016. https://www.niid.go.jp/niid/ja/allarticles/surveillance/2270-idwr/nenpou/8563-kako2017.html, Accessed 14 Feb 2020.

[32] Tao Y (2020). A nationwide spatiotemporal analysis of syphilis over 21 years and implications for prevention and control in China. Clin. Infect. Dis..

