# Supplementary material for: Phylogenetic and genetic characterization of Treponema pallidum strains from syphilis patients in Japan by whole-genome sequence analysis from global perspectives
Source: Sci Rep. 2021 Feb 4;11:3154. doi: 10.1038/s41598-021-82337-7 (PMC7862685; doi:10.1038/s41598-021-82337-7)
Supplement: Supplementary file 1 — Supplementary Figure S1. [file 41598_2021_82337_MOESM1_ESM.pptx]

## Slide 1
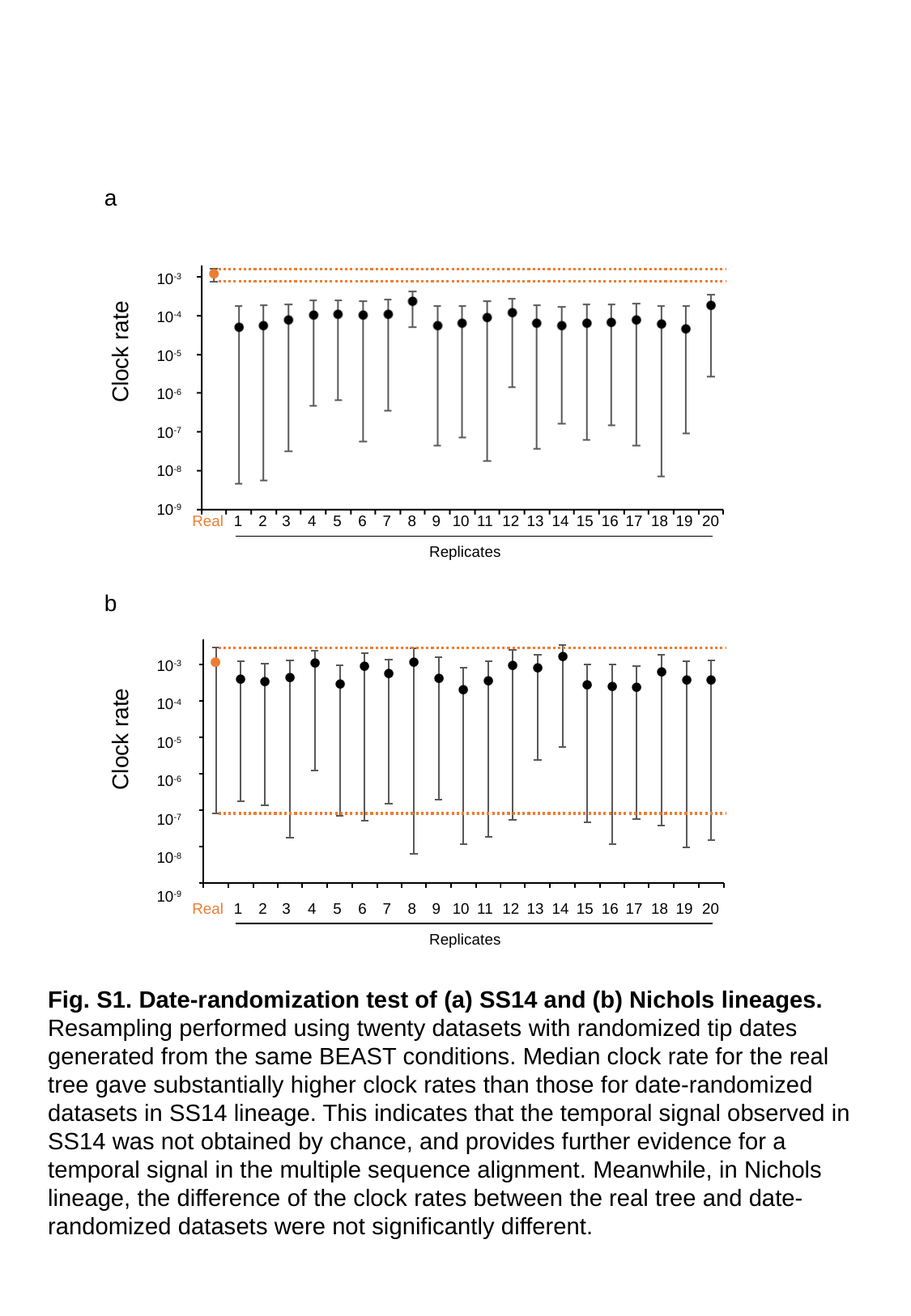

a
10-3
10-4
Clock rate
10-5
10-6
10-7
10-8
10-9
Real
1
2
3
4
5
6
7
8
9
10
11
12
13
14
15
16
17
18
19
20
Replicates
b
10-3
10-4
Clock rate
10-5
10-6
10-7
10-8
10-9
Real
1
2
3
4
5
6
7
8
9
10
11
12
13
14
15
16
17
18
19
20
Replicates
Fig. S1. Date-randomization test of (a) SS14 and (b) Nichols lineages.
Resampling performed using twenty datasets with randomized tip dates generated from the same BEAST conditions. Median clock rate for the real tree gave substantially higher clock rates than those for date-randomized datasets in SS14 lineage. This indicates that the temporal signal observed in SS14 was not obtained by chance, and provides further evidence for a temporal signal in the multiple sequence alignment. Meanwhile, in Nichols lineage, the difference of the clock rates between the real tree and date-randomized datasets were not significantly different.
